# The Cost of Perceived Overqualification: How Self-Control Depletion and Self-Leadership Shape Nurses’ Negligence Behavior

**DOI:** 10.3390/healthcare14213653

**Published:** 2026-10-28

**Authors:** Benedict Ehizojie Akhigbe, Kolawole Iyiola

**Affiliations:** Institute of Graduate Research and Studies, University of Mediterranean Karpasia, Mersin 33010, Turkey

**Keywords:** perceived overqualification, self-control depletion, self-leadership, negligence behavior, nurses

## Abstract

**Background/Objectives**: Nurses play a critical role in healthcare delivery, where their behavior can have important consequences for nursing care and patient safety. Despite growing interest in perceived overqualification, the literature remains silent on its relationship with negligence behavior among nurses, and on the mechanisms and factors that may explain or mitigate this relationship. Grounded in the conservation of resources theory (COR) and social cognitive theory, the current research examines the association between perceived overqualification (PO) and negligence behavior, focusing on self-control depletion as a mechanism and self-leadership (SL) as a moderating factor. **Methods**: Data collected through a structured questionnaire survey of 428 nurses across various hospitals in Nigeria were used to examine these relationships, which were tested using the PROCESS macro. **Results**: PO is positively related to self-control depletion and negligence behavior. Self-control depletion positively relates to negligence behavior and mediates the direct link between PO and negligence behavior. SL moderated the direct association between PO and negligence behavior, with nurses with higher self-leadership showing weaker positive relationships than those with lower self-leadership. The indirect association through self-control depletion declined at higher SL. **Conclusions**: Our findings offer new insights into how healthcare organizations can better align nurses’ qualifications with their job responsibilities, and suggest that supporting self-leadership warrants evaluation as a strategy for reducing negligence behavior.

## 1. Introduction

Negligence behavior among nurses has attracted growing academic attention and represents an emerging area of interest in healthcare systems [1,2]. Globally, it has emerged as a significant concern in healthcare due to its adverse impact on quality of care and patient safety. For instance, approximately 14% of medical errors are due to nurses’ negligent behavior [3]. Moreover, in a survey of nursing students, negligence was perceived as the most common cause of medical errors [4]. Based on this, emerging studies emphasize the importance of understanding the antecedents of negligent behavior [1,5], but no attention has been given to how nurses’ own sense of having more experience, skills, and education than their job actually requires, or that their qualifications are not utilized [6], may be related to their behavior on the job. Negligence behavior refers to a decline in an individual’s focus, aspiration, and attention at the workplace or a rise in errors when completing job tasks [7].

In the current research, negligence behavior is conceptualized as a passive, low-effort pattern of conduct in which nurses fail to perform their required job duties sufficiently (i.e., reduced work effort) and allow their effort on assigned duties to lapse or leave tasks unfinished [7]. Negligence behavior differs from several related constructs like withdrawal behavior, which involves psychological or physical removal from the job role, such as absenteeism or lateness [8]. Further, unlike counterproductive behavior, which involves actions that harm or are intended to harm [9], negligence is passive and does not require intent to harm. Moreover, negligence behavior occurs while the individual is present. Accordingly, negligence behavior is conceptualized as general work behavior that may compromise the quality of nursing care. Importantly, negligence behavior as conceptualized here is a general work behavior drawn from the employee behavior literature [1,7,10]. It is distinct from clinical negligence [11]. Therefore, the current research does not measure patient outcomes or clinical errors. Hence, its implications concern nurses’ general work conduct, which may be relevant to patient safety.

Despite the recent advancements, negligence behavior in the nursing profession remains underdeveloped regarding its antecedents [1]. Regarding its antecedents, the current literature considers only organizational factors, such as leadership [1], workplace ostracism [10] and organizational politics [12], and how these organizational or workplace factors influence negligence behavior. What is specifically unexplored and ignored is how an individual-level construct such as perceived overqualification (PO) relates to nurses’ negligence behavior.

PO is defined as an individual’s perception that their work experience, education, and skills are not used or required in their job assignments [6]. Over the past decade, PO has attracted increasing attention in organizational studies due to its adverse consequences for individual behaviors and attitudes such as reduced work engagement and job satisfaction. For meta-analytic evidence, see [13]. Further, globally, empirical evidence indicates that approximately one in three employees perceive themselves as overqualified in their current positions [13,14]. A similar pattern has been observed among nurses in the United States based on evidence of overqualification [15]. Prior studies further highlight that overqualification leads to enhanced dissatisfaction, underutilization of human capital, and higher turnover [16]. Despite the increasing focus on perceived overqualification, this phenomenon has attracted limited theoretical and empirical attention within the healthcare context [17]. Moreover, evidence from the nursing workforce indicates that nurses’ acquired competencies and skills are frequently underused, giving rise to perceptions of overqualification and underemployment [18]. This is of particular concern because such mismatch may undermine patient safety and the quality of healthcare delivery. Considering this void in the literature, this study aims to examine whether and how perceived overqualification is associated with negligence behavior.

Although emerging studies are devoting efforts to investigating the role of PO on employee outcomes [17], there remains a significant research gap in exploring how perceived overqualification translates into negative employee behaviors [9,19] such as negligence. Thus, focusing on the direct consequences (i.e., specific behavior) may lead to an inaccurate or incomplete comprehension of the action tendencies of PO [19]. One mechanism worth investigating is self-control depletion, which is defined as a state where an individual experiences a reduction in willpower and cognitive resources following prolonged self-regulation, resulting in a self-control resource shortage [20]. Given that self-control is crucial for sustaining safe practices, depleted self-regulatory resources may enhance the possibility of counterproductive behaviors [21]. Nurses who perceived themselves as overqualified may allocate more self-regulatory resources in coping with the feelings of frustration and underutilization, leaving fewer available for maintaining attentive and compliant patient care [17]. Thus, this study examines self-control depletion as a mediating mechanism that may account for the association between PO and negligence behavior.

Furthermore, considering the prevalence of perceived overqualification and unfavorable consequences [8,17], a critical question surfaces: what can individuals and organizations do to minimize the undesirable outcomes of perceived overqualification? Specifically, PO has been linked to an array of undesirable individual outcomes; little is known about the factors that buffer its adverse consequences, particularly in healthcare settings. While previous research has identified different buffering factors such as relaxation experience at work [8] and praise from patients [17], little attention has been given to personal resources that individuals can actively develop and apply in their daily work. To fill this gap, the current study examines self-leadership as a potential buffering factor that can mitigate the undesirable consequences of perceived overqualification. Self-leadership equips individuals with cognitive and behavioral strategies to regulate their emotions, thoughts, and actions, allowing them to cope more effectively with workplace challenges [22]. By investigating the moderating role of SL, this research can inform interventions and training to improve patient care and safer nursing practice.

**Figure 1:**
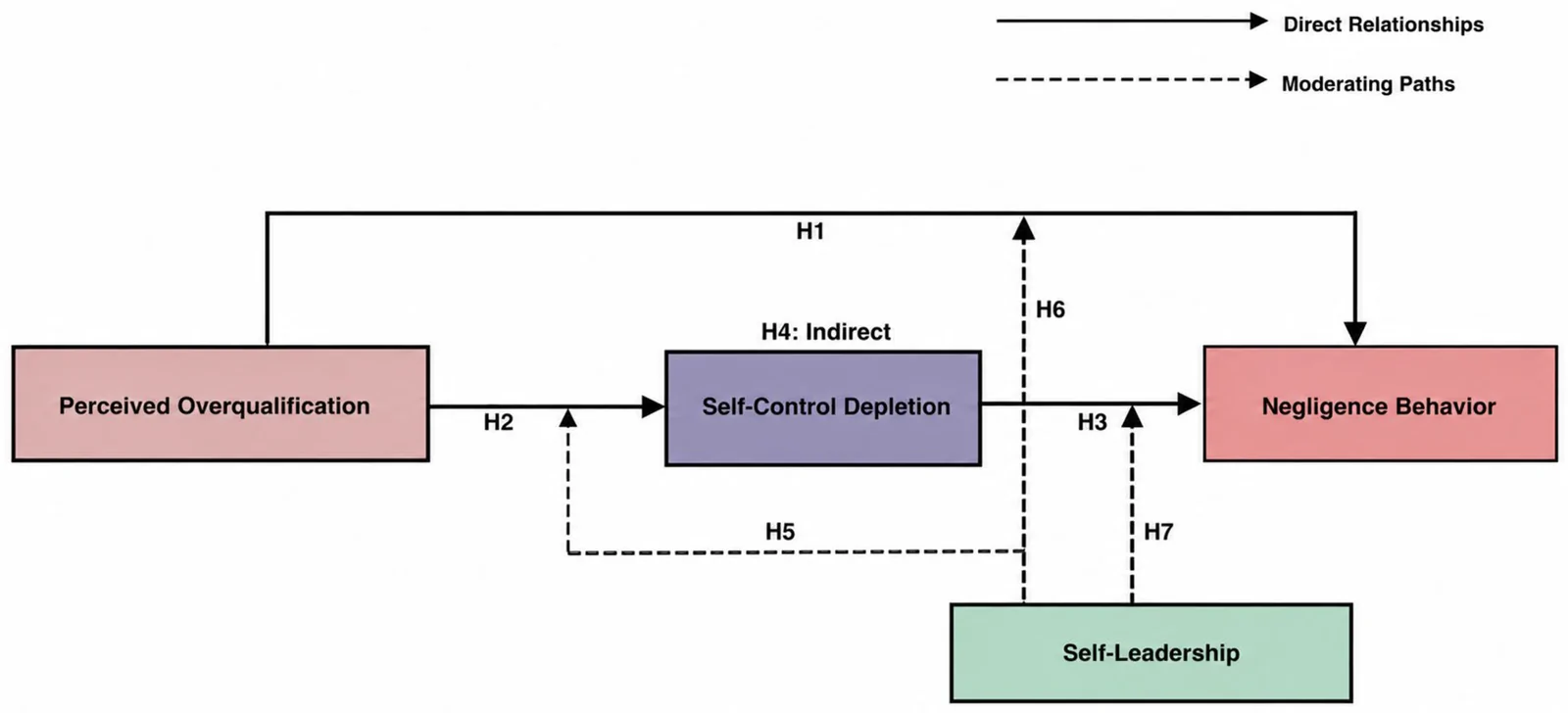
Research framework.

Drawing from conservation of resources (COR) [23] and social cognitive theory (SCT) [24], this study develops and examines an integrated research framework (depicted in Figure 1). Specifically, this research examines the association between PO and negligence behavior through the indirect role of self-control depletion. Additionally, this study examines SL as a buffering factor that can weaken positive relationships, as demonstrated in Figure 1.

Altogether, this study makes several contributions. First, using the data collected from registered nurses from hospitals in Nigeria, the current study aims to extend emerging studies in the healthcare setting [17] by uncovering the connection between PO and negligence behavior among nurses. Second, although there have been studies investigating the link between PO and its employee consequences [17], the literature lacks research on how this relationship develops. The current study introduces self-control depletion as a potential intervening variable that could help explain how perceived overqualification is related to negligence behavior. Accordingly, the current study aims to offer new insights by examining whether self-control depletion accounts for the association between perceived overqualification and negligence behavior. Third, this study responds to research calls [8,25] to explore buffering factors that minimize PO’s influence on individual outcomes. By investigating the buffering role of self-leadership, this research aims to extend the understanding of the conditions under which adverse perceived consequences of overqualification can be minimized, highlighting the potential value of self-leadership for nurses’ work conduct.

### 1.1. Theoretical Background

#### 1.1.1. Underlying Theories

The COR framework proposes that individuals strive to acquire, preserve, and protect valued resources, such as emotional, psychological, and cognitive resources, and that stress occurs when these resources are under threat, lost, or insufficient to satisfy the demands placed on them [23]. As a result, individuals experiencing resource loss become more susceptible to psychological strain and make effective work functioning harder to sustain [26]. In this study, our interest is primarily on resource loss. Within this framework, and in this study, PO is regarded as a form of person–job incongruence that threatens individual psychological resources by creating feelings of frustration and underutilization [6]. To cope with such experiences, individuals allocate sufficient resources to manage and sustain effective work behavior [27]. Based on this, recent empirical studies highlight the importance of resource-based theories such as COR [10,17] in explaining individual behaviors. Within the COR framework, continuous resource investment without sufficient replenishment ultimately depletes self-regulatory capacity [23]. Moreover, as depletion escalates, individuals become less capable of regulating their behavior, and when self-regulatory resources are taxed, later behavior becomes harder to control [28].

Another theory is the SCT [24], which is well-documented in healthcare settings [29]. SCT offers a well-grounded framework to assess how one’s actions and their consequences are related to personal, cognitive, and behavioral factors [30]. SCT proposes that individuals’ objectives drive them to conduct themselves in alignment with their goals [24]. The theory integrates cognitive and behavioral perspectives to explain human behavior. Self-leadership integrates self-regulatory characteristics by enabling individuals to set meaningful goals, monitor their thoughts, employ constructive thinking strategies, and maintain intrinsic motivation [31]. Based on this, SCT can be used to explain how self-leadership allows individuals to self-regulate and prevent depleted resources or strains from translating into undesirable behaviors. Accordingly, this study relies on these two theories to offer new insights into how and under what conditions perceived overqualification shapes negligence behavior in healthcare settings.

#### 1.1.2. Perceived Overqualification and Negligence Behavior

Although having excess qualifications may represent valuable human capital, individuals who view themselves as overqualified often experience underutilization, frustration, and lack of meaningful opportunities to apply their skills [6,13]. These unfavorable experiences have been linked to several adverse job outcomes, such as organizational commitment, turnover intentions, and counterproductive behavior [8,16,32]. Within the COR framework, when individuals have surplus qualifications, their experience, knowledge, and skills cannot be totally utilized. This will not only waste individuals’ resources but also impede their ability to generate additional resources for themselves [33]. In this context, individuals will think their resources have been lost [17]. Consistent with the COR theory, this perceived loss induces psychological strain and demands constant investment of self-regulatory resources to cope with feelings of disappointment and frustration. From this standpoint, as resource loss intensifies, individuals become more vulnerable to behavioral dysfunction and are less capable of maintaining effective work performance [34].

In the nursing profession, patient care mandates continuous vigilance, attention, and strict compliance with standards of nursing care [11]. The loss of psychological resources may have critical behavioral consequences. In this vein, nurses who perceive that qualifications are underutilized may slowly withdraw, invest lower effort in job obligations, and become less attentive to nursing care [35]. It is reasonable to infer that such a reduction in psychological investment could raise the possibility of negligence behavior. Moreover, the COR framework posits that such behaviors signify adaptive reactions to resource loss because individuals preserve their remaining resources by reducing engagement and efforts in intensive tasks [23].

Furthermore, the extant literature has demonstrated that PO is positively related to withdrawal behavior, counterproductive behavior, and work engagement [8,35]. Because negligence behavior similarly represents impaired behavioral investment and poorer compliance with expected job standards, it is anticipated that nurses who perceive themselves as overqualified are likely to engage in negligent workplace behavior. Thus, this study proposes the following:

**H1.**  *PO is positively related to negligence behavior.*

PO and self-control depletion:

Self-control depletion reflects the manifestation of the resource-loss process. Perceived overqualification, as an internal mental state because of unmet aspirations and needs associated with skill utilization in their current roles, may trigger pervasive psychological outcomes [36]. Consistent with the COR theory, individuals strive to obtain, protect, preserve, and maintain valuable resources, including emotional, cognitive, and psychological resources [23]. From this standpoint, when these valuable resources fail to produce expected returns or are threatened, individuals experience psychological strain and are forced to use additional resources to cope with such demands [37]. Despite this, the exact relationship between perceived overqualification and self-control depletion remained unresearched, particularly in the healthcare setting.

Within the COR theory, the act of forcing attention and suppressing frustration in response to demands requires a constant investment of self-control resources. Nonetheless, because overqualified individuals are not obtaining sufficient compensatory resources (external reward or task mastery) [38], they may experience net drain on their resource reservoir. Furthermore, nurses are required to consistently demonstrate self-control when providing nursing care, managing emotional strain, and upholding healthcare delivery standards [36]. From this standpoint, for nurses who view themselves as overqualified, the extra effort required to cope with constant feelings of underutilization may accelerate depletion of self-regulatory resources.

Furthermore, prior studies demonstrate that perceived overqualification contributes to burnout and emotional exhaustion [36,39]. Grounded in the COR framework, this study anticipates that continuous investment of psychological resources necessary to manage perceived overqualification undermines nurses’ self-regulatory abilities. Accordingly, perceiving oneself as overqualified feeds into the depletion of self-control. Thus, the following is posited:

**H2.**  *Perceived overqualification is positively related to self-control depletion.*

Self-control depletion and negligence behavior:

Self-control is critical for individuals to maintain focus, suppress inappropriate responses, and consistently execute tasks that demand vigilance and discipline [40]. In the nursing context, self-regulatory competencies are critical, as nurses are required to maintain professional conduct, make accurate decisions, and remain responsive to patient needs under demanding working conditions [18]. An early study conceptualized self-control as a depletable regulatory resource [41]. A central premise of COR theory is that individuals are inherently motivated to conserve their resources [23]. Within this framework, entering a state of resource loss or shortage triggers a protective psychological posture envisioned to prevent further resource depletion. In such a depleted condition, individuals’ motivational stance drifts away from resource acquisition, such as undertaking discretionary behavior [42] or striving for high performance [43]. Based on this, self-control depletion may feed nurses’ negligence behavior.

COR theory suggests that employees who have experienced resource loss adopt a defensive posture and conserve their remaining resources by reducing effort they put in demanding situations [23]. In the nursing context, several safety-relevant activities, such as monitoring patients and double-checking medications, require repetition and vigilance. Such activities may therefore be particularly vulnerable to lapses when nurses’ regulatory resources are depleted.

Furthermore, prior studies have demonstrated that self-control depletion impairs decision-making and task performance and increases errors and behavioral lapses [44,45]. In the nursing profession, where effective self-regulation is paramount to nursing care, it is anticipated that depleted self-control fosters careless work behaviors and may hinder nurses’ ability to maintain nursing care standards, increasing the likelihood of negligence behavior. Hence, we posit the following:

**H3.**  *Self-control depletion is positively related to negligence behavior.*

Self-control depletion as a mediator:

Self-control is an inherent characteristic of human behavior, and it is critical to success in life [28]. Without question, one of the most useful and important adaptations to the human psychological system is the ability to exercise self-control [46]. However, contrary to the belief that self-control is indestructible and constant, self-control is a finite resource, and it is vulnerable to certain factors [47]. Within the COR framework, self-regulatory measures deplete valuable psychological resources [23]. As resources are depleted, individuals become more vulnerable to self-control depletion, which is marked by reduced ability to regulate emotions, thoughts, and behaviors [28]. Despite this, self-control depletion as a bridging mechanism via which perceived overqualification is transmitted to nurses’ negligence behavior remained unresearched.

Perceived overqualification is considered stressful and undesirable [8,17]. Moreover, PO leads to adverse affective reactions [36,39]. Individuals experience frustration when faced with challenges and obstacles in their work environment, leading to unfilled motivations and adverse emotions [1] that can be aided by perceived overqualification. Within the COR theory, individuals experience strain when they perceive a threat to future resources or when existing resources are being depleted [23]. As mentioned earlier, perceived overqualification represents an unpleasant and stressful stimulus that depletes individuals’ key resources. Gradually, perceived overqualification may result in adverse emotions and a perception of threatened psychological or depleted resources. Hence, this study argues that nurses are prone to develop an adverse emotional state in response to perceived overqualification, which may manifest in self-control depletion.

When self-control resources are depleted, nurses are left with diminished psychological resources to manage emotional and cognitive demands of nursing care. Within the COR framework, once nurses feel self-control resources are depleted, it minimizes their passion and enthusiasm for the job [23]. With the presence of these negative emotions, nurses may experience difficulties due to this depletion in performing work tasks optimally. Moreover, nurses experiencing insufficient psychological resources are motivated to adopt coping strategies to avoid additional resource loss [23]. In this context, nurses with insufficient psychological resources are likely to protect their remaining resources by minimizing the effort and attention they invest in work activities. In this case, negligence behavior may represent the consequence of insufficient psychological resources to effectively meet the requirements of nursing care. Thus, we posit the following:

**H4.**  *Self-control depletion mediates the link between PO and negligence behavior.*

SL as a moderator:

SL functions as a self-regulation process that enables individuals to regulate their actions, interpersonal dynamics, and how they decide to lead themselves by employing behavioral and cognitive approaches [48]. To achieve this, they rely on these three behavioral and cognitive strategies to improve personal effectiveness: (1) constructive thinking strategies, (2) natural reward strategies, and (3) behavior-focused strategies [48]. In the current research, the emphasis is particularly on these strategies, given that they are related to self-management [31]. Congruent with the study’s measure, which captures all three strategy types, self-leadership is examined in this study as a global self-regulatory capability rather than a single strategy [49]. Behavior-focused strategies enhance awareness of one’s conduct, constructive thought strategies facilitate the reinterpretation of challenging situations, and natural-reward strategies help sustain motivation [50]. Together, these strategies may help individuals manage demanding work assignments and proactively direct their behavior in work contexts [50].

SL confronts the traditional belief in organizational behavior and psychology that effective leadership requires more than one person [50]. SL reconceptualizes leadership as a mechanism of self-influence, contending that individuals can proficiently serve as both leaders and followers through self-influence [51]. Based on this, several empirical studies have demonstrated that individuals who engage in SL can control their feelings, ideas, and behaviors in work settings [31,50]. Moreover, scholars have argued that individuals can develop motivation and self-direction through this internal source of leadership that promotes their work processes [50,51]. Despite this, SL, as an individual condition that may buffer the link between PO and negligence behavior, remains unresearched. Accordingly, the current research investigates self-leadership as a buffer that may either weaken or strengthen the relationships as depicted in this study’s research framework (Figure 1).

SCT can explain the mechanism through which self-leadership is expected to operate. Congruent with SCT, employees regulate their behavior through three interrelated subfunctions: self-observation of their own conduct, evaluative judgment of that conduct against personal standards, and self-reactive incentives, such as self-dissatisfaction and self-satisfaction that sustain effort even when external rewards are absent [52]. Self-leadership strategies are practical applications of these subfunctions [49]. Accordingly, constructive thought strategies might help nurses reappraise perceived overqualification by focusing on the meaningful aspect of patient care. This cognitive reframing may minimize the need to suppress frustration and conserve self-regulatory resources, minimizing the relationship between perceived overqualification and self-control depletion. Additionally, self-observation, self-goal setting, and self-reward may enable nurses to monitor lapses in their conduct and sustain efforts via self-generated incentives when external recognition is lacking. These strategies are therefore expected to weaken the extent to which perceived overqualification and depletion are expressed in negligent conduct.

Consistent with the SCT, self-led individuals can apply a series of specialized cognitive and behavioral strategies to regulate their behaviors to enhance productivity and activate proactive behaviors [53]. From this standpoint, the current research argues that nurses with high levels of SL are more likely to cognitively reframe the experience or perception of PO by focusing on the intrinsic aspects of their job and retain confidence in their performance capabilities despite perceiving a disparity between their qualifications and job expectations. Moreover, by employing regulatory techniques, such individuals can more effectively maintain motivation and manage frustration to conserve their psychological resources [31]. Accordingly, it is anticipated that nurses with high levels of self-leadership would expend fewer self-regulatory resources when managing perceived overqualification than those with low self-leadership, minimizing the possibility of experiencing self-control depletion.

Aside from conserving self-regulatory resources, congruent with the SCT, prior studies have suggested that self-leadership boosts adaptive work behaviors by activating self-evaluation, self-monitoring, and behavioral regulation [22,53]. Along these lines, nurses who engage in SL can continuously monitor their own conduct and consciously control their behaviors toward desired outcomes. On the other hand, nurses who seldom engage in self-leadership may be less likely to control their behavior when faced with dissatisfaction from perceived overqualification, making them more vulnerable to negligence behavior.

Lastly, consistent with the SCT and as discussed earlier, self-led individuals are better at regulating their feelings, emotions, and cognitions amid the perception of overqualification. This study anticipates that self-leading nurses are less likely to experience depletion, consequently interrupting the psychological process through which perceptions of overqualification transmit into negligence behavior. Hence, we posit the following:

**H5.**  *Self-leadership moderates the positive link between PO and self-control depletion, with the relationship weaker for nurses with higher self-leadership than for those with lower self-leadership.*

**H6.**  *Self-leadership moderates the positive link between PO and negligence behavior, with the relationship weaker for higher SL nurses than for those with lower SL.*

**H7.**  *The mediation of self-control depletion is moderated by self-leadership, such that with a higher level of self-leadership, the mediation impact of self-control depletion becomes weaker.*

## 2. Methods

This quantitative study used a time-lagged survey design approach to collect data from nurses working in hospitals in Lagos and Abuja, Nigeria. Hospitals in Lagos and Abuja were purposively selected because they employ a diverse and large nursing workforce. Moreover, these cities have a high concentration of public and private healthcare facilities that serve a diverse patient population.

The sampling and recruitment procedure involved two stages. In the first stage, a sampling frame of public and private hospitals in Lagos and Abuja was established using the Nigerian Health Facility Registry (HFR). Purposive sampling was used to select hospitals listed in the Nigerian HFR that operated as private and public hospitals and provided direct patient-care services involving registered nurses. Subsequently, eligible hospitals were approached for permission, leading to 238 hospitals participating in the study.

In the second stage, nurses constituted the survey units and were recruited from the participating and selected hospitals based on the following inclusion criteria: (1) nurses with a registered license; (2) in full-time employment in public or private hospitals; (3) working in a direct patient-care setting; and (4) willing to participate in the research. This inclusion strategy is common in the literature (e.g., [54]). Exclusion criteria included those who were not qualified as “nurses” as defined by the Nursing and Midwifery Council of Nigeria (NMCN) or who lacked active registration with the NMCN at the time of the research. Moreover, this research recruited only nurses on duty on the day of the survey.

This research was approved by the Ethics Committee of the University of Mediterranean Karpasia. Before data collection, the researchers obtained administrative authorization from the management of the participating hospitals to access and recruit eligible nurses. Voluntary participation, informed consent, and anonymity were guaranteed. Data collection took place between July and August 2025. After obtaining approval from the management of the sampled hospitals, a member of the research team administered the self-assessment surveys to nurses who voluntarily participated in the study and collected the completed questionnaires on site. The survey also collected information on demographic variables such as marital status, educational level, gender, age, working units or wards, and working experience.

Given the issue of common method bias in survey-based studies, this study follows the [55] approach by administering the survey and collecting data at different time intervals. This procedure involves introducing a temporal gap in data collection to minimize the respondents’ ability to remember and use prior responses as cues when answering subsequent questions [55]. Accordingly, at Time 1 of data collection, the respondents provided data on demographic information, perceived overqualification, self-control depletion, and self-leadership. The participants were contacted again a month later to provide their responses on negligence behavior at T2. A unique code identifier was assigned to the respondents who completed the survey at T1. At T2, these codes were used to match participants, and those who did not complete the survey at T1 were not allowed to participate at T2.

Overall, 788 questionnaires were administered. However, this study received only 449 useable responses from the survey nurses. Across both waves, this study obtained 428 useable matched responses (response rate of 54.31%). Moreover, this study adopted a month-long temporal separation as informed by the perceived overqualification literature (see [56]). The final sample of 428 nurses comprised 229 from Lagos and 199 from Abuja.

A power analysis using G*Power 3.1.9.7 indicated that a minimum of 395 respondents was required to detect a small-sized effect (f^2^ = 0.02) in a multiple linear regression, assuming α = 0.05 and 0.80% statistical power [57]. The obtained sample of 428 exceeded the minimum required sample size.

The sample characteristics are presented in Table 1. The sample consisted of female (333, 77.80%) and male (95, 22.20%) nurses. The respondents were split into age groups as follows: 18–25 (29, 6.78%), 26–35 (196, 45.79%), 36–40 (177, 41.36%), and over 40 (26, 6.07%). The majority of the respondents, 201 (46.97%), were married. The majority of the respondents, 359 (83.88%), had at least a bachelor’s degree. The respondents were split according to their working units as follows: surgical (174, 40.65%), medical (159, 37.15%), and others (95, 22.20%). Moreover, the majority of the respondents, 416 (97.20%), had at least 5 years of working experience.

### 2.1. Measures

PO was measured with 8 items adopted from a nine-item scale [58]. The instrument measures participants’ perceptions that their work experience, education, skills, knowledge, and abilities exceed the requirements of their current job. A sample item is “I have job skills that are not required for this job”. One item was dropped because its loading fell well below the 0.60 threshold.

Self-control depletion was measured with 4 items adopted from [59]. The scale measures the degree to which participants feel their self-control resources have been depleted. An item is “I feel like my willpower is gone.”

Self-leadership was measured with 8 items adopted from the Abbreviated Self-Leadership items [60]. The scale captures the degree to which participants use self-influence to regulate and motivate their thoughts and behaviors in pursuit of effectiveness. A sample item is “I talk to myself (out loud or in my head) to help me deal with difficult problems.”

Negligence behavior was measured with 4 items adapted from [61]. The scale measures participants’ behavioral lapses, including reduced work effort and reduced attention to responsibilities. A sample item is “I am sometimes careless in my work”. Accordingly, higher scores indicate more frequent lapses in attentiveness and work effort.

All items were rated on a five-point Likert scale (1 = strongly disagree to 5 = strongly agree).

### 2.2. Data Analysis

This study conducted data analysis using SPSS 30 and AMOS 28. Before performing descriptive and inferential analyses, the measurement items were assessed for normality, outliers, and missing values. Normality was assessed using skewness and kurtosis. The variance inflation factor (VIF) was used to assess multicollinearity in the collected data before data analysis.

Confirmatory factor analysis (CFA) was used to assess the reliability and validity of the measures prior to the testing of the proposed relationships. Hayes’ PROCESS [62] was used to test the proposed hypothesis. Specifically, Model 4 (direct and mediation) and Model 59 (moderation) were used to test the proposed relationships. The direct and indirect effects were computed using a bootstrapping procedure with 5000 resamples. The moderation analysis examined the significance of the conditional direct and indirect relationships across different levels of self-leadership (low and high). Consistent with [62], the bootstrap technique was employed to evaluate the statistical significance of the conditional indirect effect.

Furthermore, Model 59 was selected for the moderation analysis because it specifies that self-leadership moderates the first stage of the indirect path (perceived overqualification on self-control depletion; H5), the direct path (perceived overqualification on negligence behavior; H6), and the indirect association (H7). In Model 59, perceived overqualification was entered as the independent variable (X), self-control depletion was entered as the mediator (M), self-leadership as the moderator (W), and negligence behavior as the outcome (Y).

### 2.3. Non-Response Bias and Common Method Bias (CMB)

This study follows [63]’s procedure for non-response bias by using age differences to compare early and late respondents. The whole sample was divided into two groups (the first group represents the early group and the second group represents the late group). Both the early and late groups show no significant difference in participants’ age, with a t-value of 0.603 and a *p*-value of 0.547. Hence, non-response bias is not a serious concern in this study.

Because the study used self-reported data, multiple measures were adopted to minimize potential influences of CMB, including social desirability bias and perceptual distortions. The measures were adopted from previously validated scales and subjected to expert review by scholars and experts to ensure clarity, content validity, and contextual appropriateness. Further, the order of questionnaire blocks was randomized to prevent primacy and order bias [55]. For statistical control, this study adopted the marker variable approach [64]. With this approach, a theoretically unrelated variable (i.e., the marker variable) was included in the survey [65]. Accordingly, “preference for variety in leisure activities” was added to the survey. The marker variable is not theoretically and directly related to the proposed research framework. This study’s assumption was confirmed with the inclusion of the marker variable, as the model showed no significant F-change with its inclusion. Additionally, the correlation between the marker variable and the study’s core constructs was very low (0.04), indicating that CMB is not a serious threat in this study [65].

Furthermore, no single factor accounted for the majority of the total variance among the extracted factors with eigenvalues exceeding 1.0. Specifically, Harman’s single-factor test revealed that the largest factor explained 33.71% of the total variance, falling well below the conventional 50% threshold, thereby suggesting that CMB does not pose a significant threat in this study [55].

### 2.4. Reliability and Validity

Confirmatory factor analysis (CFA) was used to assess the reliability and validity of the measures. Cronbach’s alpha (α) and composite reliability (CR) were adopted to examine the reliability of the measures. According to the results depicted in Table 2, α values (0.795–0.903) and CR values (0.806 and 0.904) exceeded their respective minimum criteria of 0.6 and 0.7 [66]. Collectively, these results demonstrate the reliability of all measured constructs.

**Figure 2:**
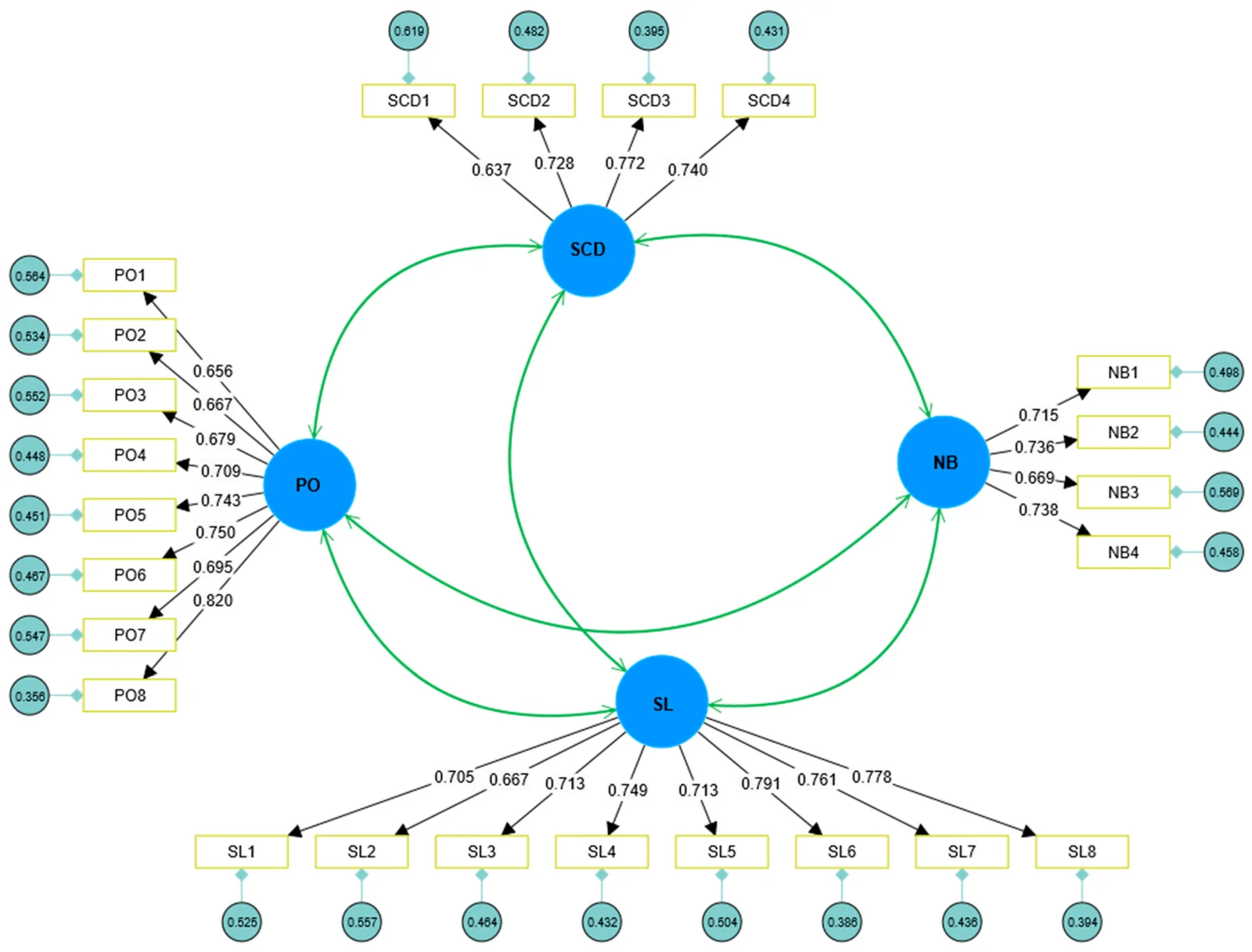
Standardized factor loadings.

To assess convergent validity, we analyzed the item-level factor loadings alongside the average variance extracted (AVE) (see Table 2 and Figure 2). The standardized loadings were from 0.656 to 0.820, satisfying the >0.60 criterion outlined by [67]. Additionally, AVE values ranged from 0.511 to 0.541, which exceeds the 0.50 cutoff. Accordingly, the convergent validity of the measures is well established [67].

This study confirmed the measurement model’s discriminant validity by applying the framework developed by [68]. This approach requires that the square root of a construct’s average variance extracted (√AVE) remains greater than its correlation with other nearby variables. The results (Table 3) meet this requirement, demonstrating that all √AVE are higher than the respective inter-construct correlation coefficients. Beyond the discriminant validity, the proposed theoretical model exhibited a strong fit to the sample data. The summary of fit indices in Table 4 (χ^2^/df = 1.085; RMSEA = 0.027; NFI = 0.927; TLI = 0.993; CFI = 0.994; GFI = 0.913; IFI = 0.994) collectively validates the model’s adequacy.

Assessment of univariate normality was ensured using the skewness and kurtosis metrics (see Table 2). With skewness values ranging from −0.210 to 0.162 and kurtosis values ranging from −0.665 to −0.220, all indicators fell comfortably within the permissible limits of |2.3| established by [69]. Consequently, the assumption of normality for the collected sample was established.

### 2.5. Hypothesis Testing: Direct and Mediation Analysis

This study adopts Model 4 of the PROCESS macro [60] to examine direct and indirect relationships. Specifically, H1 proposes that PO positively influences negligence behavior. As depicted in Table 5, the results reveal that PO is significantly and positively associated with negligence behavior (β = 0.278, *p* < 0.001), thereby validating H1. H2 predicted that PO is positively and significantly related to self-control depletion (β = 0.401, *p* < 0.001), and was supported. H3 proposes that self-control depletion positively influences negligence behavior (β = 0.289, *p* < 0.001). Further, H4 proposes that self-control depletion mediates the link between PO and negligence behavior. Further, to ensure robustness, the bootstrapping procedure was used to obtain the most reliable results for the mediation analysis. The results revealed that self-control depletion (indirect effect = 0.116, 95% CI [0.057, 0.179], not including zero) partially mediates the association between PO and negligence behavior. Based on this, H4 is supported. Moreover, the indirect effect accounts for 29.44% of the total effect.

### 2.6. Hypothesis Testing: Moderation Analysis

Model 59 of [62] was used for the moderation analysis. Before the analysis, all continuous variables were mean-centered. Gender, age, and unit/ward were also included as covariates. Table 6 presents the results of the moderation analysis. H5 proposes that the interaction between PO and SL on self-depletion control is weaker for individuals with higher self-leadership. Table 6 shows that the interaction between PO and self-leadership on self-depletion control (β = −0.010, SE = 0.049, t = −0.197, *p* > 0.05, 95% CI [−0.107, 0.088] including 0), indicating that SL did not moderate the link between PO and self-control depletion. Accordingly, H5 is not validated.

**Figure 3:**
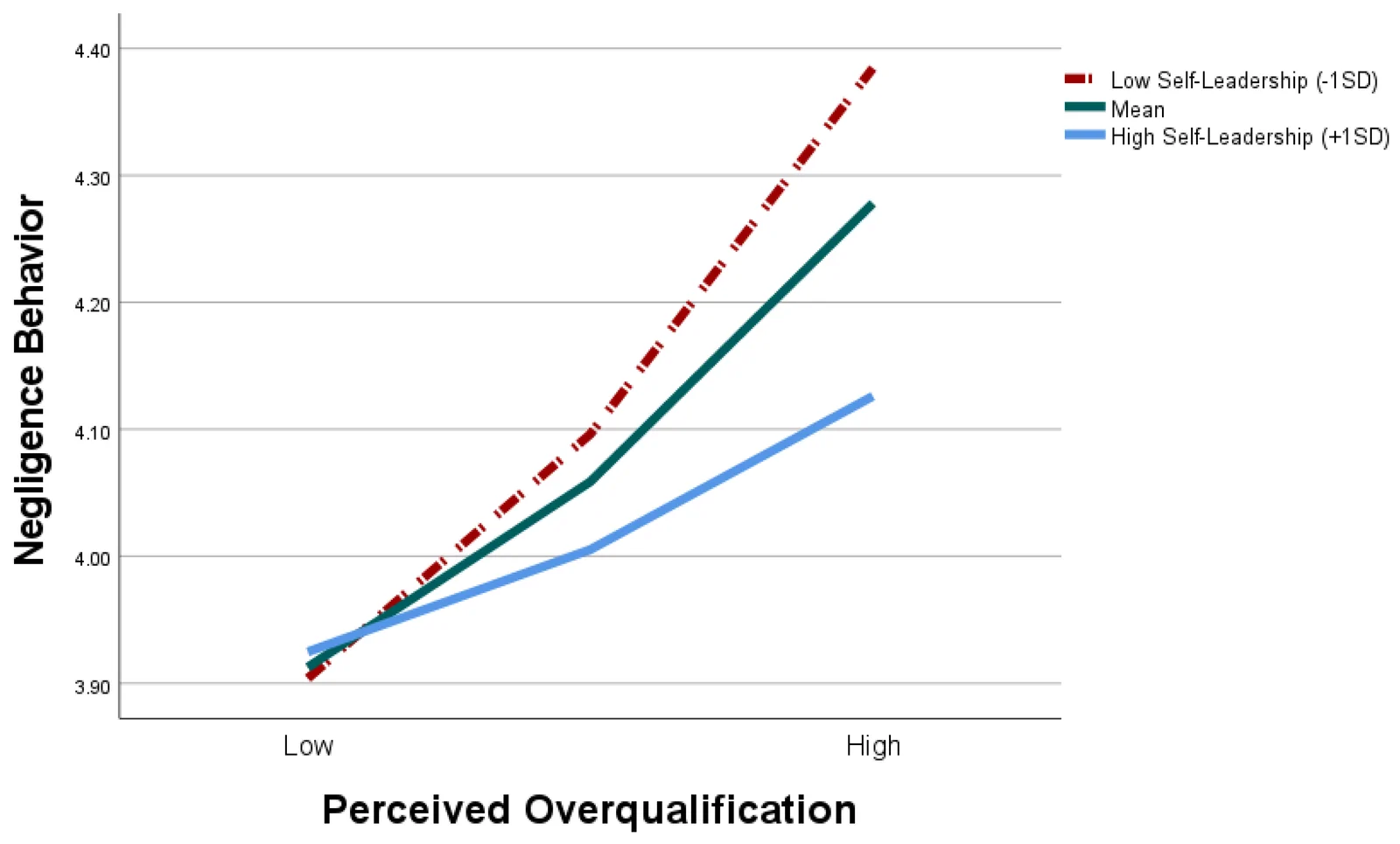
The plot of the interaction between PO and self-leadership on negligence behavior.

H6 proposed that self-leadership moderates the direct link between PO and negligence behavior. Table 6 shows that the direct link between PO and negligence behavior is significant (β = 0.280, SE = 0.047, t = 5.866, *p* < 0.001, 95% CI [0.186, 0.374]), with the direct relationship being moderated by the interaction between PO and self-leadership (β = −0.105, SE = 0.051, t = −2.057, *p* < 0.05, 95% CI [−0.206, −0.005]), validating H6. More clearly, this study adopts a simple slope approach [70] by assessing two types of means for the interaction effect (i.e., standard deviation (+1 SD), mean, and the other below the standard deviation (−1 SD)). The interaction relationship is depicted in Figure 3. Specifically, PO had a weaker positive relationship with negligence behavior in the presence of higher levels of self-leadership +1 SD (β = 0.161, SE = 0.084, t = 1.923, *p* > 0.05, 95% CI [−0.004, 0.326]) and mean (β = 0.293, SE = 0.047, t = 6.282, *p* < 0.001, 95% CI [0.201, 0.384]). Conversely, the relationship is stronger in the presence of low self-leadership (β = 0.384, SE = 0.060, t = 6.388, *p* < 0.001, 95% CI [0.266, 0.503]), showing that the link between PO and negligence behavior is stronger for nurses with low self-leadership. Thus, support is found for H6. Hence, the positive association between perceived overqualification and negligence behavior weakens as self-leadership increases, validating its proposed buffering role.

**Figure 4:**
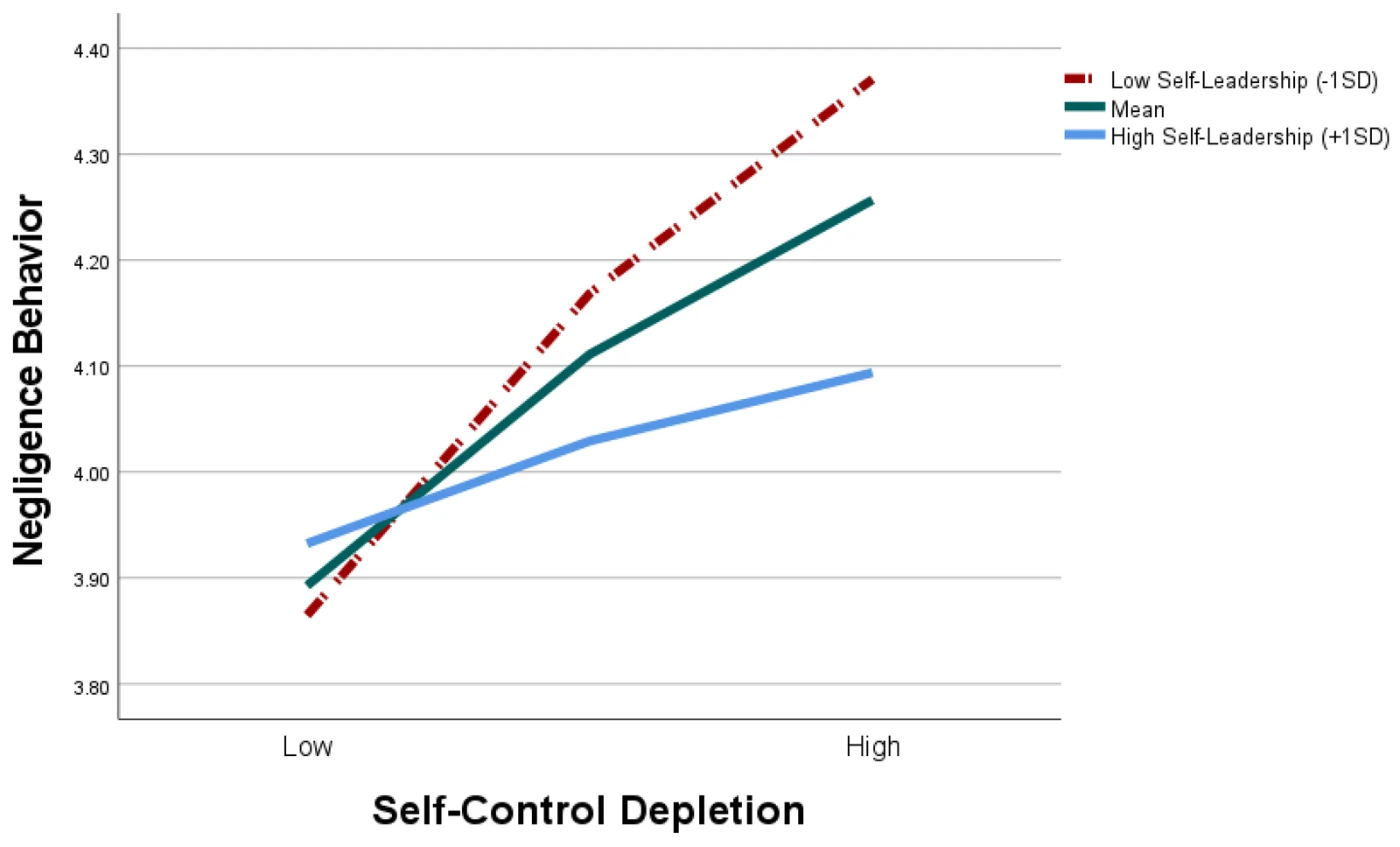
The plot of the interaction of the link between PO and negligence behavior through self-control depletion at different levels of self-leadership.

Finally, H7 predicts that SL moderates the indirect link between perceived overqualification and negligence behavior through self-control depletion. The results show that the interaction between self-leadership and PO was indirectly related to negligence behavior through self-control depletion. Specifically, for nurses with high SL, the indirect relationship was weaker (conditional indirect effect = 0.038, 95% CI from −0.019 to 0.114), whereas for nurses with low SL, the indirect relationship was significantly stronger (conditional indirect effect = 0.127, 95% CI from 0.054 to 0.220). The conditional indirect effect is depicted in Figure 4. Because a difference in significance between conditional indirect effects is not by itself a test of moderated mediation. This study further tested differences in indirect effects at high and low levels of self-leadership. The contrast was −0.090 (BootSE = 0.062, 95% bootstrap CI [−0.212, 0.030]). As hypothesized, the contrast is negative, but zero lies within its CI. Taken together, the significant second-stage moderation and the decline in the indirect effect in the hypothesized direction provide some support for H7.

## 3. Discussion

This research developed an integrated theoretical framework explaining how and when PO relates to negligence behavior among nurses in Nigeria. As proposed, PO is positively related to negligence behavior. Self-control depletion not only positively influences negligence behavior but also serves as a mechanism through which PO influences negligence behavior. SL did not moderate the link between PO and self-control depletion. However, for nurses with high SL, the direct positive link between PO and negligence behavior is weaker than that of those with low self-leadership. Moreover, SL moderates the indirect link between PO and negligence behavior through self-control depletion, with the indirect link being weaker for nurses with high SL than for those with low SL. These findings present critical implications for safer nursing practice and healthcare delivery research.

### 3.1. Theoretical Contributions

This study significantly extends the literature and contributes to the existing body of knowledge. While prior studies have explored the consequences of perceived overqualification in different industries [13,16,19,71], there are only limited efforts that have explored this concept in the healthcare setting [17,18]. At the same time, prior studies have demonstrated that perceived overqualification affects work engagement in different occupational settings in both positive and negative ways [72,73]. Despite this, little is known regarding how overqualified nurses respond to this concept in their behaviors that compromise safe nursing care. Thus, the current research extends the overqualification literature in healthcare settings by demonstrating how and when perceived overqualification is associated with nurses’ negligence behavior.

Beyond the established consequences of PO, such as turnover intentions, job dissatisfaction, counterproductive behaviors, and commitment [8,16,32], this study identifies nurses’ negligence behavior as an overlooked behavioral consequence. These prior studies mainly explored how overqualification influences employees’ well-being, attitudes, and general work-related behaviors. However, this research adopts the perspective that understanding the consequences of perceived overqualification warrants greater attention to work behaviors that may be relevant to the quality of nursing care. In doing so, this research provides new insights into the implications of overqualification for nurses’ workplace behavior. Hence, this study deepens the PO literature by showing that nurses’ perceptions of being overqualified are associated with behavioral outcomes beyond attitudinal and performance outcomes [6,38], therefore providing a more comprehensive understanding of how perceived overqualification may manifest in nursing care.

The findings suggest that PO is positively related to self-control depletion. This finding supports prior studies that perceived overqualification is related to affective and self-regulatory outcomes, such as cynicism, anger, and surface acting, [8,74]. Consistent with the COR framework, the discovery of a positive association between perceived overqualification and self-control depletion suggests that feeling overqualified is not only a cognitive appraisal of underutilization but also a resource-demanding experience that consumes nurses’ capacity for self-regulation.

Although emerging studies have demonstrated the role of PO in counterproductive behaviors [8,17], the specific association between PO and self-control depletion has not been firmly established. The literature has not offered clear insights into this specific relationship. By linking PO to self-control depletion and exploring its mediating role in nurses’ negligence behavior, the current research extends the literature by offering a more thorough understanding of the association between PO, self-control depletion, and negligence behavior. Precisely, by establishing self-control depletion as the intervening mechanism, the current research extends COR theory by demonstrating that perceived overqualification can initiate a resource-depletion process that ultimately manifests as negligence behavior. To the best of our knowledge, the existing literature has not documented the mediating role of self-control depletion on the link between PO and negligence behavior. By offering empirical insights into this indirect association, this study responds to and closes the research voids identified by [8,17].

Furthermore, and perhaps most notably, the findings of this research help clarify the nature of the relationships between perceived overqualification, self-control depletion, and negligence behavior. Surprisingly, this study found no support for the moderating role of self-leadership on the link between perceived overqualification and self-control depletion. However, the findings suggest that the behavioral consequences of PO depend on nurses’ capacity to regulate their thoughts and behaviors. Within the SCT, self-led individuals can apply behavioral and cognitive strategies to activate proactive behaviors [53]. The finding that SL weakens the positive association between PO and negligence behavior among nurses with high SL more than among those with low SL advances SCT by showing that nurses’ self-regulatory capabilities can constrain the translation of adverse work-related cognitions into dysfunctional behavior.

Moreover, recently emerging studies have begun exploring the negative intervening processes that link overqualification to its outcomes [8,16]. This study goes a step further, extending SCT by demonstrating that self-leadership operates beyond the perceived overqualification-negligence relationship and is associated with differences in the strength of the indirect process. Because self-leadership did not moderate the association between perceived overqualification and self-control depletion, moderation of the indirect association could only occur at the second stage. From this standpoint, the association between self-control depletion and negligence behavior was weaker at higher levels of self-leadership. Interpreted within SCT, this pattern suggests self-leadership may help regulate how nurses express depletion in their conduct, rather than blocking depletion itself. The indirect association also declined in the hypothesized direction. Hence, this research moves beyond whether perceived overqualification is linked to negligence behavior to uncover when its associations are less pronounced and why. Lastly, by identifying self-leadership as a self-regulatory buffer, this study shows that nurses’ personal regulatory capabilities may be relevant to the extent to which perceived overqualification becomes consequential for nurses’ work conduct.

### 3.2. Implications for Practice

In practice, negligence behavior among nurses is a critical concern for hospital management, doctors, and scholars due to its direct impact on patient health outcomes [10]. To contribute to this literature and inform managerial practice, this research developed an integrated framework explaining how nurses’ negligence behavior develops and identified pathways to minimize this dysfunctional behavior.

First, perceived overqualification has been recognized as “a detrimental work condition”, in which nurses perceive that their competencies, skills, and knowledge are underutilized in their current roles [75]; in this current research, it is positively associated with nurses’ negligent behavior. Hospital management may therefore recognize that perceived overqualification may be relevant not only to nurses’ work experiences but also to the quality of their work conduct. Hence, instead of treating overqualification mainly as an issue of job dissatisfaction and retention [16,32], hospital management may recognize that perceptions of underexploited expertise may be related to how nurses execute their responsibilities. For this reason, hiring and placement decisions could consider not only whether nurses have the necessary qualifications but also whether the scope and complexity of assigned duties offer sufficient opportunities to use those qualifications. Moreover, where notable overqualification exists, hospital administrators can minimize its negative consequences by offering mentoring, professional development, and career progression opportunities that enable nurses to apply their capabilities meaningfully.

Second, as this study’s findings indicate, perceived overqualification may be a source of self-regulatory depletion among nurses. While nursing research has linked overqualification to psychological strain and burnout, e.g., [39], the present research suggests that the consequences of overqualification may extend beyond general psychological strain to undermine nurses’ ability to regulate their emotions, thoughts, and impulses. Therefore, hospital administrators may consider measures to ease self-control depletion. Such interventions could include counseling and psychological support, such as mental health services and stress management training, to help nurses maintain and replenish their self-regulatory resources. Additionally, supervisors may integrate periodic assessment of nurses’ perceived overqualification and psychological fatigue into supervisory and performance-development practices. By addressing the resource-draining process through which perceived overqualification operates, hospitals may be better positioned to preserve nurses’ self-regulatory capacity and minimize the likelihood that depleted self-control will lead to dysfunctional behaviors.

Third, this study suggests that self-leadership may be relevant to how perceived overqualification relates to negligence behavior. Hence, this should inform health organizations that developing nurses’ self-leadership is a feasible way to reduce negligence behaviors among nurses. Therefore, hospital management may consider self-leadership as a potentially modifiable competency that could be addressed through educational programs, coaching, continuing professional development, or organizational interventions [76]. For instance, structured exercises in self-goal setting, self-observations, constructive self-talk, and the evaluation of beliefs and assumptions [49]. However, such self-reinforcement interventions should complement, rather than act as a substitute for, efforts to improve qualification–job alignment.

### 3.3. Study’s Limitations and Future Research Directions

Like other empirical research, this study has certain limitations that offer avenues for future studies. First, the nature of the survey design constrains the establishment of causal inferences. Future studies could address this by adopting a longitudinal approach to establish causal relationships. Second, although this research adopted both procedural and statistical remedies to establish CMB is not a serious concern in this study. However, this study cannot entirely rule out social desirability bias. Accordingly, future research may collect data from multiple sources (patients’ and supervisors’ perceptions of negligence behavior) to strengthen the study’s findings. Further, adopting an experimental design approach can further establish causal relationships. Third, the sample in this study is drawn from nurses working in the Nigerian healthcare sector, which may limit the generalizability of the study’s findings to other countries and sectors. Future research may replicate this study’s integrated research framework in other national cultures and sectors to establish the generalizability of the study’s findings to other contexts. Moreover, because this study was conducted in the Nigerian context, it is unclear whether the findings are representative of nurses’ behaviors across different national contexts and can be applied to global work environments. Hence, more studies are needed to establish if such behaviors are applicable to global work environments.

Fourth, based on the sampling approach adopted by this study, nurses were nested within 238 hospitals. The small number of respondents per hospital and unmeasured hospital-level factors, such as hospital type, workload, and staffing levels, may have influenced the results; staffing levels, in particular, were not measured and could not be controlled. Thus, a multi-level design with sufficient participants per hospital is recommended.

Finally, future research can extend this study’s integrated theoretical framework by investigating other antecedents of nurses’ negligence behavior. Building on this study, examining other potential direct relationships and underlying mechanisms would advance the understanding of negligence behavior literature in nursing practice. Further, the current research identified SL as a moderator in our research framework. Exploring other individual differences, such as role knowledge and identities, may further our understanding of these relationships.

## 4. Conclusions

Using a time-lagged survey of nurses in Nigeria, this study found that perceived overqualification was positively associated with self-control depletion and negligence behavior. Self-control depletion partly accounts for the direct association between perceived overqualification and negligence behavior. Self-leadership did not moderate the association between perceived overqualification and negligence behavior. However, among nurses reporting high self-leadership, the direct association between perceived overqualification and negligence behavior, and the association between self-control depletion and negligence behavior, were weaker. Moreover, the indirect association also declined at higher levels of self-leadership.

For nursing practice, the results suggest that aligning nurses’ qualifications with their roles and supporting nurses’ self-regulatory resources may be a relevant strategy to reduce lapses in work conduct.

## Figures and Tables

**Table 1:** Sample information.

Characteristics (n = 428)	Frequency	Proportion (%)
*Gender*		
Male	95	22.20
Female	333	77.80
*Age*		
18–25	29	6.78
26–35	196	45.79
36–40	177	41.36
Over 40	26	6.07
*Marital status*		
Single	152	35.51
Married	201	46.97
Others	75	17.52
*Educational level*		
College degree	69	16.12
Bachelor’s degree	302	70.56
Master’s degree	57	13.32
*Unit/Ward*		
Surgical	174	40.65
Medical	159	37.15
Others ^a^	95	22.20
*Years of experience*		
<5	12	2.80
5–10	169	39.49
10–15	142	33.18
>15	105	24.53

^a^ = Emergency unit, intensive care unit, outpatient unit, operation room.

**Table 2:** Measurement model.

Construct	Coding	FL	Cronbach’s Alpha (α)	CR	AVE	Skewness	Kurtosis
Perceived overqualification			0.893	0.894	0.514		
	PO1	0.656				−0.071	−0.451
	PO2	0.667				−0.053	−0.489
	PO3	0.679				0.007	−0.448
	PO4	0.709				0.144	−0.237
	PO5	0.743				0.039	−0.226
	PO6	0.750				0.127	−0.665
	PO7	0.695				−0.088	−0.438
	PO8	0.820				0.082	−0.586
Self-control depletion			0.809	0.812	0.520		
	SCD1	0.637				0.059	−0.375
	SCD2	0.728				−0.007	−0.573
	SCD3	0.772				−0.210	−0.413
	SCD4	0.740				0.057	−0.345
Self-leadership			0.903	0.904	0.541		
	SL1	0.705				0.029	−0.647
	SL2	0.667				0.019	−0.368
	SL3	0.713				0.067	−0.220
	SL4	0.749				0.127	−0.523
	SL5	0.713				−0.017	−0.426
	SL6	0.791				−0.006	−0.451
	SL7	0.761				0.003	−0.537
	SL8	0.778				−0.046	−0.500
Negligence behavior			0.795	0.806	0.511		
	NB1	0.715				−0.011	−0.588
	NB2	0.736				0.021	−0.389
	NB3	0.669				−0.106	−0.476
	NB4	0.738				0.162	−0.514

Note: AVE = average variance extracted, PO = perceived overqualification, SCD = self-control depletion, SL = self-leadership, NB = negligence behavior, CR = construct reliability, FL = factor loadings.

**Table 3:** Discriminant validity.

Construct	Mean	Std	PO	SCD	SL	NB	Gen	Age	Unit
PO	3.902	0.682	**0.717**						
SCD	4.097	0.671	0.407 **	**0.721**					
SL	2.994	0.877	0.366 **	0.371 **	**0.736**				
NB	4.010	0.664	0.404 **	0.407 **	0.134 **	**0.715**			
Gender	-	-	0.041	0.020	0.073	0.047	-		
Age	-	-	0.054	0.079	0.080	0.046	0.018	-	
Unit	-	-	−0.048	−0.015	−0.050	−0.011	−0.019	0.038	-

Note: std = standard deviation; ** = *p* < 0.01; values in bold are √AVE.

**Table 4:** Summary of model fit.

Fit Index	Acceptable Fit Threshold	Model Values	Model Fit Assessments
Chi-Square/Degree of Freedom (χ^2^/df)	≤3	1.085	Excellent
Root Mean Square Error of Approximation (RMSEA)	≤0.08	0.027	Excellent
Normed Fit Index (NFI)	≥0.9	0.927	Acceptable
Tucker–Lewis Index (TLI)	≥0.9	0.993	Excellent
Comparative Fit Index (CFI)	≥0.9	0.994	Excellent
Goodness of Fit Index (GFI)	≥0.9	0.913	Acceptable
Incremental Fit Index (IFI)	≥0.9	0.994	Excellent

**Table 5:** Estimation of the mediation model.

Path	Coefficient			Model Summary		
	β	SE	t	R^2^	F	ρ
	**Model 1**					
Perceived overqualification → self-control depletion	0.401	0.044	9.198	0.165	84.607	0.000
	**Model 2**					
Perceived overqualification → negligence behavior	0.278	0.045	6.140	0.234	65.042	0.000
Self-control depletion → negligence behavior	0.289	0.046	6.281			0.000
	**Testing mediation**					
	**β**	**BootSE**	**t**	**LLCI**	**ULCI**	**Effect sizes (%)**
Total effect	0.394	0.043	9.118	0.309	0.479	100
Direct effect	0.278	0.045	6.140	0.189	0.367	70.56
Perceived overqualification → self-control depletion → negligence behavior (indirect effect)	0.116	0.032		0.057	0.179	29.44

**Table 6:** Estimation of moderation analyses.

Model	β	SE	t	ρ	LLCI	ULCI
**Mediation model analysis: Self-control depletion as response**						
Intercept	−0.098	0.134	−0.732	0.464	−0.360	0.165
Perceived overqualification	0.305	0.046	6.507	0.000	0.213	0.398
SL	0.196	0.036	5.456	0.000	0.125	0.267
**H5**: Perceived overqualification × self-leadership	−0.010	0.049	−0.197	0.843	−0.107	0.088
Gender	−0.018	0.069	−0.254	0.799	−0.155	0.119
Age	0.039	0.040	0.961	0.337	−0.041	0.118
Unit	0.009	0.037	0.255	0.798	−0.064	0.083
R^2^	0.225					
F	20.333					
ρ	0.000					
**Dependent model analysis: Negligence behavior as response**						
Intercept	4.035	0.129	31.217	0.000	3.782	4.289
Perceived overqualification	0.280	0.047	5.866	0.000	0.186	0.374
Self-control depletion	0.276	0.049	5.569	0.000	0.178	0.373
SL	−0.046	0.036	−1.263	0.207	−0.117	0.025
**H6 Interaction**: Perceived overqualification × SL	−0.105	0.051	−2.057	0.040	−0.206	−0.005
**H7 Interaction**: Self-control depletion × SL	−0.129	0.056	−2.319	0.021	−0.239	−0.019
Gender	0.001	0.067	0.022	0.983	−0.131	0.134
Age	0.009	0.039	0.228	0.819	−0.068	0.086
Unit	0.001	0.036	0.034	0.973	−0.069	0.072
R^2^	0.267					
F	19.165					
ρ	0.000					
**Conditional direct effect of PO on negligence behavior at different levels of self-leadership**						
Low self-leadership	0.384	0.060	6.388	0.000	0.266	0.503
Self-leadership (mean)	0.293	0.047	6.282	0.000	0.201	0.384
High self-leadership	0.161	0.084	1.923	0.055	−0.004	0.326
**Conditional indirect effect of self-leadership:** **(perceived overqualification → self-control depletion → negligence behavior)**						
Low self-leadership	0.127	0.043			0.054	0.220
Self-leadership mean	0.089	0.023			0.047	0.138
High self-leadership	0.038	0.033			−0.019	0.114
Contrast	−0.090	−0.062			−0.212	0.030

## Data Availability

Data can be obtained from the corresponding author upon reasonable request.
